# Experimental Activation of Endocannabinoid System Reveals Antilipotoxic Effects on Cardiac Myocytes

**DOI:** 10.3390/molecules25081932

**Published:** 2020-04-21

**Authors:** Ewa Harasim-Symbor, Agnieszka Polak-Iwaniuk, Karolina Konstantynowicz-Nowicka, Patrycja Bielawiec, Barbara Malinowska, Irena Kasacka, Adrian Chabowski

**Affiliations:** 1 Department of Physiology, Medical University of Bialystok, 15-222 Bialystok, Poland; karolina.konstantynowicz@umb.edu.pl (K.K.-N.); patrycja.bielawiec@umb.edu.pl (P.B.); adrian@umb.edu.pl (A.C.); 2 Faculty of Health Sciences, Lomza State University of Applied Sciences, 18-400 Lomza, Poland; agn.polak@wp.pl; 3 Department of Experimental Physiology and Pathophysiology, Medical University of Bialystok, 15-222 Bialystok, Poland; bmalin@umb.edu.pl; 4 Department of Histology and Cytophysiology, Medical University of Bialystok, 15-222 Bialystok, Poland; kasacka@umb.edu.pl

**Keywords:** spontaneously hypertensive rats, URB597, ceramide, diacylglycerol, glucose, insulin signaling

## Abstract

Hypertension coincides with myocardial alternations in lipid (including sphingolipids) and glucose metabolism. The latest data indicate that accumulation of metabolically active lipids, especially ceramide (CER) and diacylglycerol (DAG) significantly influences intracellular signaling pathways along with inducing insulin resistance. Since, it was demonstrated that the endocannabinoid system (ECS) affects myocardial metabolism it seems to be a relevant tool in alleviating metabolic disturbances within the cardiac muscle due to hypertension. All designed experiments were conducted on the animal model of primary hypertension, i.e., spontaneously hypertensive rat (SHR) with chronic ECS activation by injections of fatty acid amide hydrolase (FAAH) inhibitor—URB597. Lipid analyses were performed using chromatography techniques (gas liquid, thin layer, and high performance liquid chromatography). Colorimetric and immunoenzymatic testes were applied in order to determine plasma concentrations of insulin and glucose. Total myocardial expression of selected proteins was measured by Western blotting and/or immunohistochemistry methods. SHRs exhibited significantly intensified myocardial de novo pathway of CER synthesis as well as DAG accumulation compared to the control Wistar Kyoto rats. Besides, intramyocardial level of potentially cardioprotective sphingolipid, i.e., sphingosine-1-phosphate was considerably decreased in SHRs, whereas URB597 treatment restored the level of this derivative. Unexpectedly, ECS upregulation protected overloaded cardiac muscle against CER and DAG accumulation. Moreover, chronic URB597 treatment improved intramyocardial insulin signaling pathways in both normotensive and hypertensive conditions. It seems that the enhanced ECS triggers protective mechanisms in the heart due to decreasing the level of lipid mediators of insulin resistance.

## 1. Introduction 

Nowadays, hypertension is one of the most common civilization diseases according to the World Health Organization. Globally the incidence of hypertension has risen dramatically since in 2017 the recommended threshold for its diagnosis was established at 130/80 mmHg (systolic and diastolic blood pressure, respectively) [1]. Importantly, chronically elevated blood pressure (BP) triggers ventricular hypertrophy and contractile impairments in cardiac muscle together with its clinical manifestations (cardiovascular diseases, CVDs). Hypertension itself causes changes in metabolism of the heart and in most cases coincides with metabolic disturbances, i.e., obesity, insulin resistance or type 2 diabetes [2,3,4]. Therefore, at present relieving health consequences of hypertension and related myocardial metabolic alternations is critical.

For this purpose a suitable and commonly used genetic model of primary hypertension, i.e., spontaneously hypertensive rats (SHRs), has been selected. These animals with age spontaneously develop hypertension, in which systolic blood pressure can reach the level of 180-200 mmHg [5]. Furthermore, it was noticed that SHRs apart from exhibiting hypertrophy and failure of the heart are also insulin resistant with decreased peripheral glucose utilization, which corresponds to metabolic pattern of hypertensive patients [6,7]. However, the precise molecular mechanism underlying this state is not yet fully understood and described. Several lines of evidence have shown that accumulation of biologically active lipids, i.e., ceramide (CER) and diacylglycerol (DAG) in insulin sensitive tissues (e.g., skeletal and cardiac muscle or liver), can interfere with intracellular pathway of that hormone (especially protein kinase B—AKT/PKB and glycogen synthase kinase 3—GSK3) and consequently impair its action on target cells [8,9,10]. This in turn may lead to the development of homeostatic imbalance in myocardial ATP production, nutrient storage and subsequently disrupt cardiac contractile activity. It is especially important in the pressure-overloaded heart. Furthermore, decreased glucose uptake in pressure overloaded cardiomyocytes may be even more detrimental, since myocardial fueling preference in hypertension is shifted towards glucose utilization rather than fatty acids [11,12,13].

Thus, new potential therapies for the treatment of hypertension and co-existing metabolic disorders are constantly being sought. In recent years the endocannabinoid system (ECS) is of great interest for such treatment due to diversity of exerted effects as well as broad expression of its two G_i/o_ protein-coupled cannabinoid receptors (i.e., CB_1_ and CB_2_) in both nervous system and peripheral tissues. Endogenous ligands (polyunsaturated fatty acid amides) of these receptors are anandamide (AEA) and 2-arachidonyl glycerol (2-AG), which are metabolized mainly by two enzymes, i.e., fatty acid amide hydrolase (FAAH) and monoacylglycerol lipase (MAGL), respectively [14]. Extensive studies have proven that this inner lipid signaling system has significant influence on the whole body by interfering energetic homeostasis, central nervous system functions, immune responses, gastrointestinal motility and blood pressure as well [15,16,17]. Moreover, we and others [18,19,20,21] have shown in animal models of primary (SHRs) and secondary (DOCA salt) hypertension that elevated tone of ECS in 2-week time frame can be a beneficial in markedly declining both systolic and mean blood pressures. Importantly, we have also reported that upregulation of ECS significantly alters intramyocardial fatty acid and glucose metabolism in hypertensive state [20,21,22]. We found that chronic activation of ECS by FAAH inhibition substantially enhances palmitate uptake and its esterification but not oxidation in the left ventricle of SHRs [20,23]. However, there are no studies revealing the link between prolonged activation of ECS in primary hypertension and insulin signaling pathway in cardiomyocytes. Taken altogether, it was reasonable to examine whether chronic activation of ECS (by FAAH inhibition and thereby increasing the level of AEA) affects cardiomyocytes’ insulin signal transmission in relation to the content of bioactive lipid species (i.e., DAG and CER). In order to achieve that objective we conducted experiments on SHRs with 2-week stimulation of ECS (intraperitoneal URB597 injections) and evaluated plasma levels of glucose and insulin, myocardial content of DAG, CER, and other sphingolipids as well as the expressions of proteins engaged in insulin signaling (e.g., AKT and GSK3) and diacylglycerol metabolism, i.e., diacylglycerol acyltransferase 1 and 2 (DGAT1,2) as well as adipose triglyceride lipase (ATGL). 

## 2. Materials and Methods

### 2.1. Animals and Experimental Model

Spontaneously hypertensive and Wistar Kyoto (WKY) rats (6–7 week old, 170–200 g) were purchased from the Center of Experimental Medicine of the Medical University of Bialystok, Poland. The animals were kept in an approved animal holding facilities (22 °C ± 2, 12 h light–dark cycle) with unrestricted access to standard pellet chow (Labofeed B, Animal Feed Manufacturer “Morawski”, Kcynia, Poland) and tap water. All experiments and procedures conducted on the rats were approved by the Ethical Comitee for Animal Experiments at the Medical University of Bialystok, Poland. 

After a period of acclimatization (1 week) the animals were randomly divided into four experimental groups, i.e., WKY—normotensive, control rats; WKY+URB597—normotensive rats treated with URB597; SHR—hypertensive rats and SHR+URB597—hypertensive rats treated with URB597 (each group included 6 rats). Prolonged activation of the endocannabinoid system in the normotensive and hypertensive conditions was acquired by chronic inhibition of fatty acid amide hydrolase by URB597 (30-(aminocarbonyl)[1,10-biphenyl]-3-yl)-cyclohexylcarbamate) treatment [18,21]. Briefly, the rats from both normotensive and hypertensive groups received intraperitoneal (i.p.) injections of URB597 (1 mg/kg of body mass) or its vehicle (1:2:7; DMSO:Tween80:0.9% NaCl) twice a day for 14 consecutive days [18,24]. In previous studies we [20,21] and others [18,19] have shown that chronic URB597 treatment considerably decreases blood pressure (BP) in a model of primary (SHRs) as well as secondary hypertension (DOCA-salt rats) [21,25,26]. Moreover, during time course of the experiment body weight, hemodynamic parameters, and heart hypertrophy were evaluated, which was published previously [20].

### 2.2. Lipid Analyses 

In the left ventricle we have measured intramyocardial content of diacylglycerols and sphingomyelin (SM) by the means of gas liquid chromatography (GLC). Briefly, samples of the cardiac muscle were powdered and lipids were extracted in a chloroform-methanol solution according to the Folch method [27]. Thereafter, DAG and SM fractions were separated by thin-layer chromatography (TLC) [28] and then individual fatty acid methyl esters were identified and quantified based on retention times of standards by GLC (Hewlett-Packard 5890 Series II gas chromatograph, HP-INNOWax capillary column). Total DAG and SM contents were calculated as the sum of the particular fatty acid species of selected fractions and the values were expressed in nanomoles per gram of tissue.

Ceramide, sphinganine (SFA), sphingosine (SFO), and sphingosine-1-phosphate (S1P) contents in the left ventricle were estimated by high performance liquid chromatography (HPLC), as it was described [29]. Briefly, samples of the cardiac muscle were homogenized and lipids were extracted into chloroform. Aliquots of the lipid extracts were transferred to a new tubes and an internal standard was added as well (40 pmol of N-palmitoyl-D-erythro-sphingosine; C17 base). Then, the samples were subjected to alkaline hydrolysis to form deacylate ceramide. Free sphinganine and sphingosine released from ceramide were converted to their o-phthalaldehyde derivatives and analyzed by HPLC system equipped with a fluorescence detector and C18 reversed-phase column (Varian Inc. OmniSpher 5, 4.6 × 150 mm).

### 2.3. Plasma Measurements

Plasma glucose and insulin concentrations were determined using Glucose Assay Kit (Abcam, Cambrigge, UK) and Rat Insulin ELISA Kit (Mercodia AB, Uppsala, Sweden), respectively. All procedures during measurements were performed according to the manufacturer’s instructions. For each measurement, calculated values were based on a separate standard curve and the intensity of colored products was measured in a hybrid multi-mode microplate reader (Synergy H1^TM^, BioTek Instruments, Winooski, VT, USA). Moreover, we evaluated insulin sensitivity index (validated in rats [30]), i.e., the homeostasis model assessment of insulin resistance (HOMA-IR), where fasting plasma glucose (FPG) concentration was expressed in milligrams per deciliter and fasting plasma insulin in microunits per milliliter (HOMA-IR = (FPG × FPI)/2.430).

### 2.4. Immunoblotting Analysis

Total protein expression was detected using Western blotting procedure, as it was previously reported [9]. Briefly, bicinchonic acid method (BCA) with bovine serum albumin (BSA) as a standard was applied in order to determine protein concentration in the myocardial homogenates. Next, proteins were separated by 10% sodium dodecyl sulphate (SDS) polyacrylamide gel electrophoresis and transferred on nitrocellulose membranes in wet conditions. Then, all the membranes were blocked in Tris Buffer Saline Tween20 (TBST) containing 5% non-fat dry milk or BSA. Next step involved overnight incubation of nitrocellulose membranes with selected primary antibodies, i.e., diacylglycerol acyltransferase 1 (DGAT1, 1:500, Novus Biologicals, USA), diacylglycerol acyltransferase 2 (DGAT2, 1:500, Santa Cruz Biotechnology, USA), adipose triglyceride lipase (ATGL, 1:1000, Santa Cruz Biotechnology, USA), glucose transporter 1 (GLUT1, 1:500, Santa Cruz Biotechnology, USA), glucose transporter 4 (GLUT4, 1:500, Santa Cruz Biotechnology, USA), insulin receptor substrate 1 (IRS-1, 1:1000; Cell Signaling Technology, UK), phosphorylated insulin receptor substrate 1 (pIRS1 (Ser302), 1:1000; Cell Signaling Technology, UK), protein kinase B (AKT/PKB, 1:1000; Cell Signaling Technology, UK), phosphorylated protein kinase B (pAKT/PKB (Ser473), 1:1000; Cell Signaling Technology, UK), glycogen synthase kinase 3 (GSK3, 1:500; Thermo Scientific, USA), phosphorylated glycogen synthase kinase 3 (pGSK3 (Ser9), 1:500, Thermo Scientific, USA), glyceraldehyde 3-phosphate dehydrogenase (GAPDH, 1:500, Santa Cruz Biotechnology, USA), mitogen-activated protein kinase (MAPK; MAPK3/1, 1:1000; Cell Signaling Technology, UK), phosphorylated mitogen-activated protein kinase (pMAPK (Thr202/Tyr204); pMAPK3/1, 1:1000; Cell Signaling Technology, UK), citrate synthase (CS, 1:500, Santa Cruz Biotechnology, USA) and pyruvate dehydrogenase (PDH, 1:4000; Abcam, UK). Afterwards, nitrocellulose membranes were washed and incubated with appropriate secondary antibodies conjugated with horseradish peroxidase (diluted in TBST). The above mentioned proteins were visualized using Clarity Western ECL Substrate (Bio-Rad, USA) suitable for chemiluminescence detection. Obtained signals were quantified densitometrically using ChemiDoc visualization system (Bio-Rad, Poland). The same concentration of protein was loaded on each lane (30 µg), which was confirmed by Ponceau S staining. Additionally, expression of each analyzed protein was standardized to GAPDH expression and the control group (WKY) was set as 100%.

### 2.5. Immunohistochemical Analysis 

Our immunohistochemical (IHC) measurements were based on the EnVision method according to Herman and Elfont [31]. Detailed procedure of IHC staining for GLUT1 (anti-GLUT1, 1:500, sc-7903, Santa Cruz Biotechnology, Santa Cruz, CA, USA) and GLUT4 (anti-GLUT4, 1:500, sc-53566, Santa Cruz Biotechnology, Santa Cruz, CA, USA), which was performed, is already described [21,22]. The results were evaluated on an Olympus BX41 microscope with an Olympus DP12 camera under 200 magnification. The images were morphometrically analyzed using NIS-Elements Advanced Research software of Nikon. The intensity of the IHC staining was evaluated using a 0 to 256 grey scale level in six randomly selected sites of each rat’s heart, in which white or bright pixels received a value of 0 and black pixels were indicated as 256 value. 

### 2.6. Statistical Analyses

All data are expressed as mean values ± SD or percentage of the control group based on six independent determinations. Statistical differences between groups were tested with two-way analyses of variance (ANOVA) or Kruskal–Wallis test and appropriate post-hoc tests using Graph Pad Prism 5 (GraphPad Software, La Jolla, USA). Values P < 0.05 were considered statistically significant.

## 3. Results 

### 3.1. Effect of Enhanced Activation of the Endocannabinoid System on Plasma Levels of Glucose and Insulin as Well as HOMA-IR in the Normotensive State and Primary Hypertension 

Plasma measurement showed that both groups of spontaneously hypertensive rats (untreated and treated with URB597) exhibited significantly higher concentration of glucose compared to normotensive control rats (+14.3% and 15.4%, P < 0.05; Table 1, respectively). In addition, chronic ECS activation considerably reduced plasma insulin level as well as HOMA-IR index in SHRs (–32.3% and –39.4%, P < 0.05; Table 1, respectively) compared to the control hypertensive rats.

### 3.2. Effect of Enhanced Activation of the Endocannabinoid System on the Sphingolipid Pathway (Sphinganine, Ceramide, Sphingosine, and Shingosine-1-phosphate) in the Left Ventricle in Both Normotensive State and Primary Hypertension

**Figure 1:**
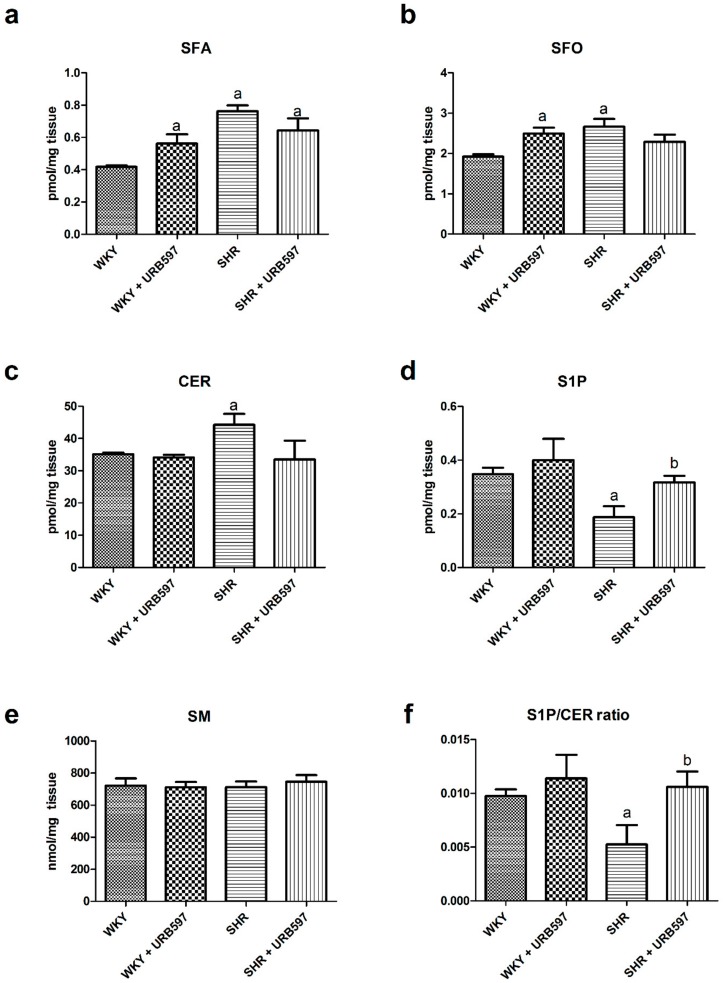
The effect of chronic URB597 treatment on the left ventricle content of sphinganine—SFA (**a**), sphingosine—SFO (**b**), ceramide—CER (**c**), sphingosine-1-phosphate—S1P (**d**), sphingomyelin—SM (**e**) and sphingosine-1-phosphate/ceramide ratio (**f**) in control Wistar Kyoto rats (WKY) and spontaneously hypertensive rats (SHRs). The data are expressed as the mean ± SD, n = 6 in each group. ^a^ P < 0.05 significant difference: control group (WKY) vs. examined group; ^b^ P < 0.05 significant difference: SHR vs. SHR + URB597.

We noticed that the intramyocardial SFA and SFO contents were significantly augmented in the normotensive rats chronically treated with URB597 compared to the control group (+34.4% and +29.7%, P < 0.05; Figure 1a,b, respectively). Interestingly, de novo pathway of ceramide formation was enhanced in SHRs since we observed an elevation in SFA (+82.3%, P < 0.05; Figure 1a), SFO (+38.6%, P < 0.05; Figure 1b) and CER levels (+26.2%, P < 0.05; Figure 1c) compared to the normotensive state. Additionally, SHR rats exhibited simultaneous decrease in S1P content (–46.0%, P < 0.05; Figure 1d) and S1P/CER ratio (–46.2%, P < 0.05; Figure 1f) in comparison with the normotensive group. On the other hand, prolonged inhibition of FAAH in SHRs substantially increased S1P level (+68.5%, P < 0.05; Figure 1d) and concomitantly S1P/CER ratio (+101.9%, P < 0.05; Figure 1f) in the left ventricle compared to the SHR group. Nevertheless, in the SHR+URB597 group it was also displayed simultaneous elevation in SFA content (+53.6%, P < 0.05; Figure 1a) compared to the control group. Both hypertension and prolonged ECS activation did not affect myocardial content of sphingomyelin (P > 0.05; Figure 1e). 

### 3.3. Effect of Enhanced Activation of the Endocannabinoid System on the Left Ventricle Content of Diacylglycerol and Total Expression of Enzymes Involved in Its Metabolism in the Normotensive State and Primary Hypertension

**Figure 2:**
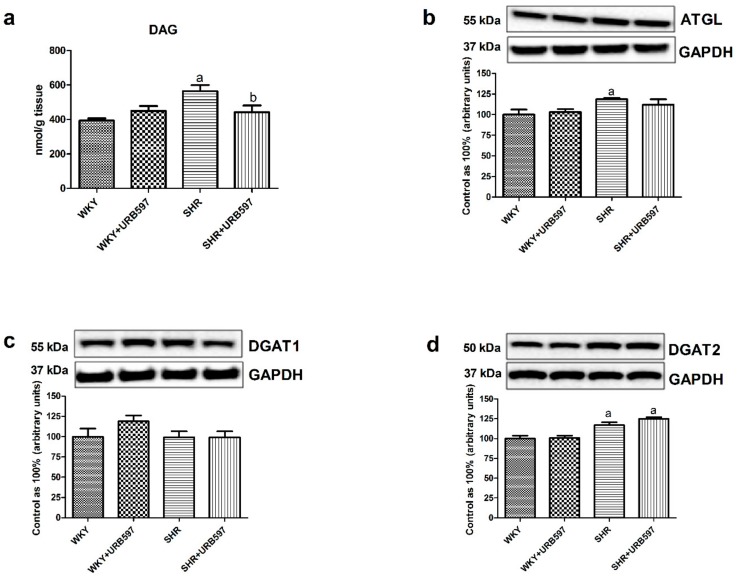
The effect of chronic URB597 treatment on the left ventricle content of diacylglycerols (DAG) (**a**) as well as total expression of adipose triglyceride lipase—ATGL (**b**), diacylglycerol acyltransferase 1—DGAT1 (**c**) and diacylglycerol acyltransferase 2—DGAT2 (**d**) in the control Wistar Kyoto rats (WKY) and spontaneously hypertensive rats (SHRs). Total expression of the above proteins was presented as a percentage difference compared to the control group, which was set as 100%. The data are expressed as the mean ± SD, n = 6 in each group. ^a^ P < 0.05 significant difference: control group (WKY) vs. examined group; ^b^ P < 0.05 significant difference: SHR vs. SHR + URB597.

The SHRs exhibited a significant increase in the myocardial DAG content (+43.6%, P < 0.05; Figure 2a) as well as total expression of ATGL (+18.6%, P < 0.05; Figure 2b) and DGAT2 (+17.0%, P < 0.05; Figure 2d) in the left ventricle in comparison with the Wistar Kyoto normotensive rats. Moreover, we observed that 2-week URB597 treatment caused a substantial reduction in cardiac DAG pool in the model of primary hypertension compared to the hypertensive group not treated with FAAH inhibitor (–21.8%, P < 0.05; Figure 2a). Concomitantly, in the same group of experimental animals there was a significant elevation of myocardial DGAT2 expression (+24.9%, P < 0.05; Figure 2d) compared to the control normotensive rats. 

### 3.4. Effect of Enhanced Activation of the Endocannabinoid System on the Total Expression and Phosphorylation of Intramyocardial (Left Ventricle) Insulin Signaling Proteins and Proteins Involved in Glucose Metabolism in the Normotensive State and Primary Hypertension

**Figure 3:**
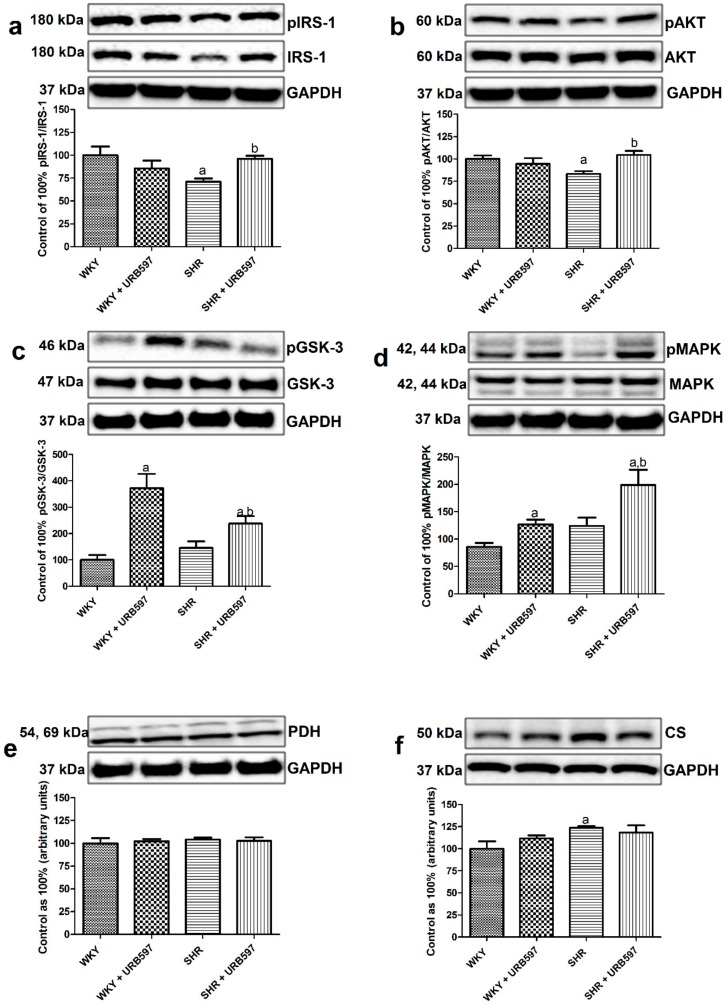
The effect of chronic URB597 treatment on the ratio of total expression of phosphorylated and unphosphorylated proteins involved in insulin signaling pathway, i.e., phosphorylated insulin receptor substrate 1/insulin receptor substrate 1—pIRS-1/IRS-1 (**a**), phosphorylated protein kinase B/protein kinase B—pAKT/AKT (**b**), phosphorylated glycogen synthase kinase 3/glycogen synthase kinase 3 pGSK-3/GSK-3 (**c**), and phosphorylated mitogen-activated protein kinase/mitogen-activated protein kinase—pMAPK/p44/42 MAPK (**d**) as well as total expression of pyruvate dehydrogenase—PDH (**e**) and citrate synthase—CS (**f**) in the left ventricle in the control Wistar Kyoto rats (WKY) and spontaneously hypertensive rats (SHRs). Calculated ratio and total expressions of the above proteins were presented as a percentage difference compared to the control group, which was set as 100%. The data are expressed as the mean ± SD, n = 6 in each group. ^a^ P < 0.05 significant difference: Control group (WKY) vs. examined group; ^b^ P < 0.05 significant difference: SHR vs. SHR + URB597.

In the experimental model of primary hypertension we observed a decrease in myocardial phosphorylation of IRS-1 (Ser302) (−28.9%, P < 0.05; Figure 3a) and AKT/PKB (Ser473) proteins (–16.9%, P < 0.05; Figure 3b) with simultaneous increase in the total CS expression (+23.8%, P < 0.05; Figure 3f) compared to the normotensive rats. Moreover, our study has demonstrated that 2-week treatment with FAAH inhibitor caused a marked elevation of IRS-1 phosphorylation (+35.2, P < 0.05; Figure 3a vs. SHR group), AKT/PKB (+25.8%, P < 0.05; Figure 3b vs. SHR group), GSK-3 (+137.6%, vs. control group and +63.2% vs. SHR group, P < 0.05; Figure 3c) and MAPK (+132% vs. control group and +60.3% vs. SHR group, P < 0.05; Figure 3d) in the hypertensive group. Furthermore, chronic URB597 treatment resulted in pronounced elevation of GSK-3 (+272.2%, P < 0.05; Figure 3c) and MAPK (+48.0%, P < 0.05; Figure 3d) phosphorylation in the SHR hearts compared to the normotensive WKY rats.

**Figure 4:**
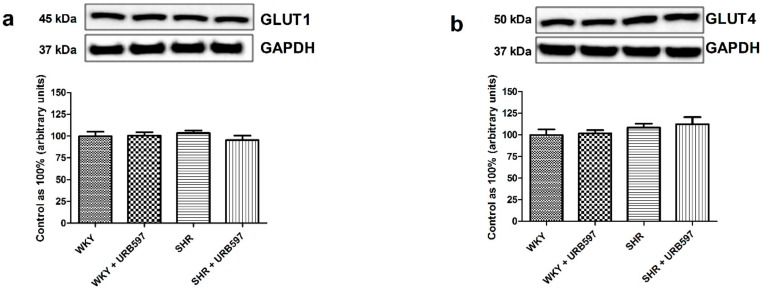
The effect of chronic URB597 treatment on total expressions of glucose transporter 1—GLUT1 (**a**) and glucose transporter 4—GLUT4 (**b**) in the left ventricle of the control Wistar Kyoto rats (WKY) and spontaneously hypertensive rats (SHRs). Total expressions of the above proteins were presented as a percentage difference compared to the control group, which was set as 100%. The data are expressed as the mean ± SD, n = 6 in each group.

**Figure 5:**
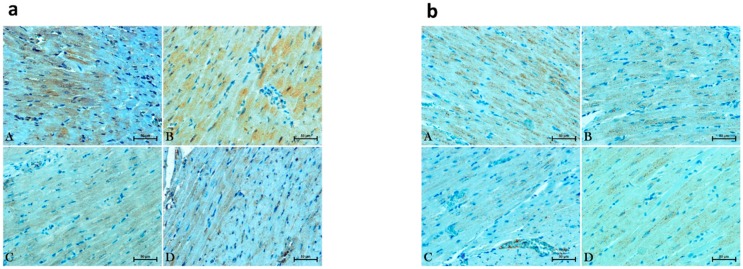
Representative images of glucose transporter 1—GLUT1 (**a**) and glucose transporter 4—GLUT4 (**b**) expressions in the left ventricular cardiomyocytes in the control Wistar Kyoto rats (WKY) and spontaneously hypertensive rats (SHRs), where (**A**) is WKY group, (**B**) WKY + URB597 group, (**C**) is SHR group and (**D**) SHR + URB597.

In parallel to the above changes we did not notice any alternations in the total myocardial expression of GLUT1 and GLUT4 (P > 0.05; Figure 4a,b and Figure 5a,b) in all the examined groups compared to the normotensive group or hypertensive group after URB597 treatment.

## 4. Discussion

Our experiment shed light on new effects exerted by chronic upregulation of ECS in relation to cardiomiocytes’ intracellular signaling pathways and lipid metabolism. This novel insight in the action of ECS seems to be relevant since previously we have found that enhanced endocannabinoid tone significantly alters cardiac muscle energetic milieu in both normotensive and hypertensive conditions [20,21,22]. It is important to note that present study, which was based on a genetic model of primary hypertension (SHRs), has revealed the link between chronic activation of ECS by URB597 treatment (2-week) and insulin signaling pathway in cardiomyocytes subjected to the increased afterload. Our data suggest that ECS upregulation ameliorates impaired myocardial insulin signal transduction in SHR rats. Nevertheless, in previous studies [21,22,32] we have reported that chronic FAAH inhibition in a model of secondary hypertension considerably elevated intramyocardial lipid and glycogen deposition along with palmitic acid uptake in order to facilitate adjustment in fuel demands of overloaded left ventricle. We provide evidence upon different routes of URB597 action in SHRs, including significant reduction of BP which corresponds with elevated palmitate uptake and its esterification as well as an enhancement in the expression of selected insulin signaling proteins (e.g., IRS-1, AKT, GSK-3, MAPK). Taken together, our experimental model also confirms the existence of a dynamic connection between cardiovascular parameters and metabolic status of the myocardium.

Current study has shown an increased intramyocardial content of DAG and CER fractions in the spontaneously hypertensive rats compared to the normotensive rats. Recent data provide several lines of evidence to indicate that the above lipid fractions, when accumulated, can trigger lipotoxicity and are implicated in intracellular signaling impairments, including insulin pathway (in cardiac and skeletal muscles or liver) [8,9,33,34]. Therefore, DAG and CER are referred as lipid mediators of insulin resistance (IR). In a number of studies it was shown that increased levels of bioactive lipid species such as DAG and CER can interfere with downstream cellular insulin transduction through dysregulation of major proteins involved in this transmission, i.e., IRS-1, AKT/PKB or atypical protein kinases C (PKC) activation [35,36]. This, in turn leads to energetic and nutrient imbalance in the heart due to reduced glucose uptake as well as glycogen storage. Furthermore, it was demonstrated that elevated contents of DAG and CER fractions together with reactive oxygen species (ROS) and other metabolites are engaged in the development of changes in the cardiac muscle’s structure, mitochondrial activity, endoplasmic reticulum stress and apoptosis [37]. Importantly, in this respect our results exhibit a probable protective role of ECS due to observed reduction in the content of DAG and lack of an increase in CER fraction in SHRs (in the presence of chronic URB597 treatment). Moreover, the above observations coincide with enhancement of insulin signaling transmission in the left ventricle compared to the control hypertensive rats. Consequently, we have noticed that prolonged activation of ECS in SHRs was parallel with markedly enhanced phosphorylation of proteins involved in the myocardial insulin action, i.e., IRS-1, AKT/PKB, GSK-3, and MAPK, which may indicate and reflect causal relationship. What is more, probably the above alternations arise from plasma measurements, which revealed that enhanced ECS tone ameliorated insulin sensitivity by reducing significantly insulin concentration in hypertensive rats. Even though, studies on the ground of ECS control over insulin secretion from pancreatic islets are still contradictory [38,39,40]. The above changes are in line with our previous investigation, where it was demonstrated that URB597 treatment had restored myocardial glycogen level in DOCA-salt hypertensive rats [22], which is also controlled by insulin. However, we have to bear in mind that the ability of SHRs to maintain energetic homeostasis (including glucose metabolism) in the cardiac tissue decreases with age [32]. Moreover, we are aware of the fact that the limitation of this study is lack of direct cardiac muscle stimulation by insulin for instance in isolated primary cardiomyocytes after chronic URB597 treatment. Nonetheless, the obtained results undoubtedly indicate causal relationship between ECS upregulation and improvement in overloaded cardiac muscle metabolic pathways, including insulin. In the study conducted by Zhang et al. [33] on mice fed a high fat diet there was a proposed mechanism by which accumulated DAGs, through translocation of protein kinase C-α to the plasma membrane and impaired IRS-1 phosphorylation, led to the development of cardiac insulin resistance. Furthermore, in our previous study [20], we have shown that chronic URB597 administration to SHRs promoted palmitic acid incorporation into neutral lipid fractions in the heart, i.e., triacylglycerol (TAG), free fatty acid (FFA) and cholesterol rather than DAG, as it was demonstrated in the present study. What is more, in the same research we revealed that chronic URB597 treatment enhances fatty acid oxidation only in normotensive conditions, which was linked with an elevation in peroxisome proliferator-activated receptor alpha (PPARα) expression, with parallel no effects in SHRs [20]. Additionally, as it was indicated in current experiments, a significant decline in the myocardial DAG content in SHRs treated with URB597 was followed by alternations in the expression of DGAT2 and ATGL, two major enzymes involved in the lipid metabolism. Therefore, we can presume that enhanced activation of ECS can serve as a cardioprotective tool due to diminishing potentially harmful lipotoxic level of DAG and CER.

As we mentioned earlier one of our goals was to examine intramyocardial level of ceramide, as a potentially detrimental lipid derivative, in a genetic model of primary hypertension. Hence, we have investigated major pathways of sphingolipid metabolism under the conditions of high BP and chronic activation of ECS. To our knowledge, for the first time we have shown that SHRs exhibit a substantially increased CER content in the heart. The increase was a result of greater de novo ceramide synthesis pathway activation, since simultaneous rise in SFA content compared to the normotensive rats was observed (with concomitant no change in SM content). Interestingly, the same group of hypertensive rats displayed a considerably lower value of S1P/CER ratio in comparison with the normotensive conditions, which may have a relationship with decreased activation of cardiomyocytes’ signaling pathways (IRS-1 and AKT/PKB) and changed apoptotic status as well. However, the most profound alternations were observed in the myocardial sphingolipid metabolism in SHRs when ECS tone was elevated. Surprisingly, 2-week URB597 treatment prevented overloaded cardiac muscle against CER accumulation and caused further increase in sphingosine-1-phosphate as well as S1P/CER ratio compared to the hypertensive rats, which may confirm protective role of ECS in the hypertensive heart in respect of lipotoxicity. Spijkers et al. [41] demonstrated that S1P/CER ratio in SHRs was shifted towards ceramide accumulation in carotid artery. Moreover, in the same study it was revealed that plasma level of CER was elevated in both SHR and hypertensive patients [41], which also correlated with progression of hypertension. Nevertheless, the above mentioned change in intramyocardial S1P level in our model of hypertension seems to be relevant due to the fact that compared to CER and SFO this sphingolipid derivative has potential cardioprotective properties [42,43]. Moreover, in the previous research it was found that S1P acts as a vasoactive substance and triggers vasodilation depending on activation of endothelial nitric oxide synthase [44,45], thereby, affecting metabolic status of the tissue.

## 5. Conclusions

In the present research we have described for the first time the link between alternations in myocardial content of ceramide and other sphingolipids, diacylglycerol as well as metabolic pathways in spontaneously hypertensive rats after chronic ECS activation. Furthermore, we have shown that prolonged FAAH inhibition (by URB597 treatment) protected against accumulation of insulin resistance lipid mediators (such as CER and DAG) in hypertensive state. Importantly, apart from the above changes ECS upregulation also overlaps with substantial improvement in phosphorylation status of major metabolic pathways (i.e., GSK-3 and AKT) in the cardiomyocytes. Even though, our experimental model has some limitations, it emphasizes a new cardioprotective approach of ECS action in spontaneously hypertensive rats.

## Figures and Tables

**Table 1:** Plasma glucose and insulin concentrations as well as the homeostasis model assessment of insulin resistance (HOMA-IR) after prolonged activation of ECS in normotensive control Wistar Kyoto rats (WKY) and spontaneously hypertensive rats (SHRs). The data are expressed as the mean ± SD, n = 6 in each group. ^a^ P < 0.05 significant difference: control group (WKY) vs. examined group; ^b^ P < 0.05 significant difference: SHR vs. SHR + URB597.

	WKY	WKY + URB597	SHR	SHR + URB597
**Glucose (mg/dL)**	103 ± 12	96 ± 10	118 ± 10 ^a^	119 ± 15 ^a^
**Insulin (μg/L)**	1.91 ± 0.13	1.52 ± 0.22	2.21 ± 0.61	1.58 ± 0.31 ^b^
**HOMA-IR**	1.14 ± 0.13	0.81 ± 0.12	1.44 ± 0.41	1.03 ± 0.21 ^b^
